# In Vitro Quantified Determination of β-Amyloid 42 Peptides, a Biomarker of Neuro-Degenerative Disorders, in PBS and Human Serum Using a Simple, Cost-Effective Thin Gold Film Biosensor

**DOI:** 10.3390/bios7030029

**Published:** 2017-07-20

**Authors:** Yifan Dai, Alireza Molazemhosseini, Chung Chiun Liu

**Affiliations:** 1 Department of Chemical & Biomolecular Engineering and Electronics Design Center, Case Western Reserve University, 10900 Euclid Avenue, Cleveland, OH 44106, USA; yxd176@case.edu; 2 Dipartimento di Chimica, Materiali e Ingegneria Chimica “Giulio Natta”, Politecnico di Milano, via~Mancinelli 7, 20131 Milan, Italy; axm1058@case.edu

**Keywords:** β-amyloid 42, differential pulse voltammetry, neuro-degenerative disorders, ferrocyanide/ferricyanide redox couple

## Abstract

A simple in vitro biosensor for the detection of β-amyloid 42 in phosphate-buffered saline (PBS) and undiluted human serum was fabricated and tested based on our platform sensor technology. The bio-recognition mechanism of this biosensor was based on the effect of the interaction between antibody and antigen of β-amyloid 42 to the redox couple probe of K_4_Fe(CN)_6_ and K_3_Fe(CN)_6_. Differential pulse voltammetry (DPV) served as the transduction mechanism measuring the current output derived from the redox coupling reaction. The biosensor was a three-electrode electrochemical system, and the working and counter electrodes were 50 nm thin gold film deposited by a sputtering technique. The reference electrode was a thick-film printed Ag/AgCl electrode. Laser ablation technique was used to define the size and structure of the biosensor. Cost-effective roll-to-roll manufacturing process was employed in the fabrication of the biosensor, making it simple and relatively inexpensive. Self-assembled monolayers (SAM) of 3-Mercaptopropionic acid (MPA) was employed to covalently immobilize the thiol group on the gold working electrode. A carbodiimide conjugation approach using *N*-(3-dimethylaminopropyl)-*N*′-ethylcarbodiimide hydrochloride (EDC) and *N*–hydroxysuccinimide (NHS) was undertaken for cross-linking antibody of β-amyloid 42 to the carboxylic groups on one end of the MPA. The antibody concentration of β-amyloid 42 used was 18.75 µg/mL. The concentration range of β-amyloid 42 in this study was from 0.0675 µg/mL to 0.5 µg/mL for both PBS and undiluted human serum. DPV measurements showed excellent response in this antigen concentration range. Interference study of this biosensor was carried out in the presence of Tau protein antigen. Excellent specificity of this β-amyloid 42 biosensor was demonstrated without interference from other species, such as T-tau protein.

## 1. Introduction

Diagnosis of neuro-degenerative disorders, particularly Alzheimer’s Disease (AD), is difficult and inconclusive. Brain imaging and biomarkers of cerebrospinal fluid (CSF) have been advocated for technical diagnosis of AD [1]. The CSF biomarkers include total tau (T-tau), hyperphosphorylated tau (P-tau), and the 42 amino acid isoforms of amyloid β (β-amyloid 42) [1,2,3]. β-amyloid 42 pathophysiology leads to plaque deposition and can also accelerate antecedent limbic and brain tauopathy [4,5,6]. Scientifically, the diagnosis of AD follows two pathways: the tauopathy and the amyloidopathy. In the tauopathy, the detection of T-tau and P-tau proteins are considered to be essential [7]. In the amyloidopathy, the measurement of β-amyloid 42 is important [8]. In practical assessment, the examination of β-amyloid 42 accumulated in senile plagues and intracellular neurofibrillary tangles of P-tau is considered the hallmark of identification of AD [2,3]. Unfortunately, the assessment of the β-amyloid 42 accumulations is often performed post mortem without providing an early and meaningful assessment of β-amyloid 42 for the progression of AD [2,7,8] and other amyloid related diseases.

Therefore, it will be advantageous to detect β-amyloid 42 and use it as a biomarker for the assessment of the progression or the pathological process at an early stage of AD. β-amyloid 42 has been used as a biomarker in CSF for AD. Detection of β-amyloid 42 has been exploited using different analytical techniques, such as mass spectrometry [9], surface plasmon resonance [10], scanning tunneling microscopy [11], electrophoresis [12,13], the enzyme-linked immunosorbent assay (ELISA) [14], and others. Detection of β-amyloid 42 by the ELISA technique is commonly used in liquid-based test samples. However, various research groups have reported that the standard ELISA test technique can give a very high measurement background, resulting in flawed and inconclusive assessment of β-amyloid [10,15,16,17]. Furthermore, the ELISA technique is elaborate and requires a skilled operator. ELISA technique provides accurate results; however, it is time-consuming and relatively expensive, compared to our platform biosensor technique. Measurement of β-amyloid 42 in CSF is valuable; however, the collection of CSF remains to be a complicate and expensive procedure. It will be more attractive and practical for β-amyloid 42 to be detected in other physiological fluids, including plasma, serum, and others. Consequently, research efforts have been devoted to detecting β-amyloid 42 in physiological fluids other than CSF [17,18,19,20,21]. Therefore, it is desirable to have a simple-use detection method for β-amyloid 42 in a physiological fluid other than CSF, such as serum and others. It is necessary that this detection technique for β-amyloid 42 has the needed sensitivity and selectivity to make the quantification of β-amyloid 42 meaningful for practical applications, such as the production, accumulation, and clearance of β-amyloid 42 under the physiological and patho-physiological scenarios [21].

Biosensors provide a potential opportunity in this advancement. Researchers have explored the use of different modified electrodes for bio-sensing applications, such as graphene and graphene-based materials, graphene hybrids, chemical doping, as well as magnetic doping materials including Fe_3_O_4_ nano-magnetic particles [22,23,24,25,26,27]. These modifications are elegant and elaborate but with limited sensor uniformity and reproducibility; calibration of individual biosensor is often required and time-consuming. Consequently, practical and extensive use of the modified sensors for β-amyloid 42 measurement remains limited.

In this study, our biosensor system for the detection of β-amyloid 42 is single-use, disposable, cost-effective, and time-efficient. Estimated cost for production of our biosensor, including antibody cost, is around $3. Further, preparation of the biosensor was completed before actual testing and the actual detection test time only requires 30 s. The bio-recognition mechanism was based on the interaction of antibody and antigen of β-amyloid 42. The effect of this interaction on a well-established redox couple reaction, Fe^+2^/Fe^+3^, as K_4_Fe(CN)_6_ and K_3_Fe(CN)_6_ in solution form, was used as the transduction mechanism in this development. Differential pulse voltammetry (DPV) measured the current output resulting from the electrochemical reaction of K_4_Fe(CN)_6_ and K_3_Fe(CN)_6_ , which was influenced by the interaction between the antibody and the antigen of β-amyloid 42. This current output can then be used to quantify β-amyloid 42 level at a known quantity of the antibody of β-amyloid 42. DPV applied a linear sweep voltammetry with a series of regular voltage pulses superimposed on the linear potential sweep. The current was then measured immediately before each potential change. Thus, the effect of the charging current could be minimized, achieving a higher sensitivity. The biosensors were manufactured by the industrial roll-to-roll process, which was very cost-effective. The thin gold film electrodes were deposited by sputtering physical vapor deposition at the atomic level. Thus, the working and counter thin gold film electrode elements were uniform and highly reproducible. In this study, a concentration range of 0.0625 µg/mL to 0.5 µg/mL of β-amyloid 42 antigens was investigated. Both phosphate buffered saline (PBS) of 0.1 M and human serum were used as the test media. The selectivity of this biosensor was examined using an interference study by the T-Tau in the concentrations of 0.125 µg/mL and 0.5 µg/mL at corresponding β-amyloid 42 antigen concentrations. The interference study results showed excellent selectivity. All measurements were carried out at room temperature (23 °C).

## 2. Materials and Equipment

### 2.1. Reagents and Apparatus

Anti-β-amyloid 1-42 (#ab180956) and recombinant human β-amyloid 1-42 protein (#ab82795) was purchased from Abcam (Cambridge, MA, USA). Tau protein ladder (#T7951) was purchased from Sigma Aldrich (St. Louis, MO, USA). Phosphate buffered saline (PBS) 1.0 M (pH = 7.4), 3-Mercaptopropionic acid (MPA), *N*-(3-dimethylaminopropyl)-*N*′-ethylcarbodiimide hydrochloride (EDC) and *N*–hydroxysuccinimide (NHS) were also purchased from Sigma-Aldrich (St. Louis, MO, USA). Concentrated H_2_SO_4_ (95.0 to 98.0 *w*/*w* %), concentrated HNO_3_ (70% *w*/*w* %) were received from Fisher Scientific (Pittsburgh, PA, USA). Human serum (Cat. # 3667), K_3_Fe(CN)_6_ and K_4_Fe(CN)_6_ (Cat. # P3289 and P3667) were obtained from Sigma-Aldrich (St. Louis, MO, USA). All the chemicals were used without further purification. A CHI 660C (CH Instrument, Inc., Austin, TX, USA) Electrochemical Workstation was used for DPV and the electrochemical impedance spectroscopy (EIS) characterization investigations.

### 2.2. Design and Fabrication of the Biosensor

This β-amyloid 42 biosensor was based on our platform sensor design and fabrication. The biosensor was a three-electrode configuration electrochemical sensor. Both the working and counter electrodes were thin gold film in 50 nm thickness, and the reference electrode was a thick-film printed Ag/AgCl electrode. The thin gold film was deposited by sputtering physical vapor deposition on the atomic level, without any binder, as done in thick film printing. Consequently, the electrodes were very uniform and highly reproducible. Laser ablation technique was used to define the design structure and size of the biosensor and each electrode elements. Nazdar APL 34 silicone-free dielectric thick film ink was used for the insulation layer defining the biosensor structure. Polyethylene terephthalate (PET) was used as the substrate for the fabrication of the biosensor. The overall dimensions of an individual biosensor were 33.0 × 8.0 mm^2^. The working electrode area was 1.54 mm^2^, accommodating 15–25 µL of liquid test sample. The manufacturing of the biosensor was accomplished on a cost-effective roll-to-roll process resulting in a cost-effective, single-use, disposable in vitro biosensor for β-amyloid 42 detection. Typically, each sheet of PET substrate (355 × 280 mm^2^) would produce 100 individual biosensors in 4 rows. More detailed explanation of the design and fabrication of this platform biosensor were reported elsewhere [28,29].

## 3. Functionalization of the Biosensor

### 3.1. Chemical Cleaning of the Biosensor

Chemical cleaning was the first step of the functionalization of the biosensor. This cleaning step eliminated any oxidized compounds and residue from the gold film electrode, minimizing the electrode charge transfer resistance, thereby enhancing the sensitivity and reproducibility of the biosensor. This process was a 3-step chemical pretreatment procedure and was based on the study by others [30,31], as well as in our own previous studies [29,32], with minor modifications. Typically, a batch of 8 thin gold film based biosensors were immersed in a 2 M KOH solution for 15min. After rinsing with copious amounts of DI water for about 30 s, the biosensors were placed in a 0.05 M H_2_SO_4_ solution (95.0 to 98.0 *w*/*w* %) for another 3–5 min. DI water was then used to rinse the biosensor prototypes for another 30 s. The biosensors were then placed in a 0.05 M HNO_3_ solution (70% *w*/*w* %) for another 3–5 min. The biosensors were rinsed one more time with DI water for 30 s and dried gently in a steam of nitrogen. EIS was employed to assess the success of this cleaning process. A solution of K_4_Fe(CN)_6_ and K_3_Fe(CN)_6_ of 5 mM in each component was prepared in 0.1 M PBS and used for EIS tests. Typically, two groups of the biosensors were used in this test. One group of the biosensors were subjected to the cleaning protocol described above, whereas another group of the biosensors were cleaned by ethanol and deionized water (DIW) sequentially. 20 µL of the redox couple, K_4_Fe(CN)_6_ and K_3_Fe(CN)_6_ solution prepared, was placed on top of the sensing area of each biosensor for the EIS test. The EIS study of the pretreated biosensors showed excellent reproducibility, identical to the results reported in our previous studies [28,29].

### 3.2. Functionalization of the Biosensor

The functionalization of the biosensor was accomplished in two steps. The first step was to establish a thiol bond, which provided an excellent affinity to the gold film electrode surface. The second step was to functionalize the carboxylic group at the other end of the thiol bond for the attachment of the antibody of β-amyloid 42. Thiol modification of the gold electrode surface used in this study is a well-acknowledged technique [33,34,35,36,37].

Self-assembled monolayers (SAM) of 3-Mercaptopropionic acid (MPA) were used in the first step of this procedure [33,34,35,36,37]. Typically, 8 biosensors were prepared in this immobilization step as a batch. 1 mM solution of 3-MPA in ethanol was first prepared and the biosensors were immersed in this solution for 24 h in the dark, then, the biosensors were rinsed with DI water and dried gently in a steam of N_2_. The second step of this functionalization process was then carried out. In this second step, the carboxylic groups on the other end of the 3-MPA-modified gold film electrodes (AuEs) were then functionalized by incubating in a 0.1 M PBS (pH = 7.4) solution containing 0.25 M EDC and 0.05 M NHS for 5 h. The activated AuEs were then rinsed by 0.1 M PBS and dried by N_2_ flow gently. 20 µL of 18.75 µg/mL anti- β-amyloid 42 was placed on the sensing area of each AuE and left to dry overnight at 4 °C. Antibody immobilized biosensors were rinsed with 0.1 M PBS and stored at 4 °C.

### 3.3. Differential Pulse Voltammetry (DPV) Measurement

Cyclic voltammetry (CV) and chronoamperometry (CA) are generally used in biomedical measurements. CV and CA provide sufficient sensitivity in practical biomedical applications. The required electronic interface for CV and CA are relatively simple. Differential Pulse Voltammetry (DPV) is a well-established electroanalytical technique [38], however, its applications to biomedical measurement has not been fully exploited. DPV applies a series of regular potential pulse superimposed on the potential stair steps. The current is then measured immediately prior to each potential change. Consequently, the charging current can be minimized, resulting in a higher sensitivity. DPV has shown effective and sensitive measurement in our previous studies of hemoglobin A1c (HbA1c), T-tau and 17 β-estradiol detections [29,32,39], and it is also used in this β-amyloid 42 study. In this study, the antibody of β-amyloid 42 was first bonded and functionalized as described above. The biosensors were then immersed in β-amyloid 42 antibody solution for 20 h at 4 °C. For testing, the antigen solutions of β-amyloid 42 were prepared both in 0.1 M PBS, and undiluted human serum. After the incubation, the biosensors were rinsed with 0.1M PBS, removing any unbounded antibody of β-amyloid 42. A solution of K_4_Fe(CN)_6_ and K_3_Fe(CN)_6_ , 5 mM in each component, was prepared in 0.1 M PBS and 20 µL of this redox probe was placed on top of the sensing area of the biosensor, and DPV measurements were then took place.

## 4. Results and Discussion

### 4.1. Preparation of β-Amyloid 42 Antigen Solutions

Self-assembly of β-amyloid 42 peptides was well-recognized in previous studies [40,41]. In order to prevent aggregation of β-amyloid 42 peptides, dimethyl sulfoxide (DMSO) was first applied for dissolving β-amyloid 42 peptides directly. Previous reports proved that DMSO solvent was effective in slowing the aggregation rate of β-amyloid peptides, and our previous study proved that DMSO has no interference with PBS solution [39]. After dissolving the peptides with DMSO, 0.1 M PBS solution or human serum was used for further dilution to different antigen concentrations. Detectable concentration range of β-amyloid 42 was determined by the structure of immunoglobulin G (IgG) type antibody used in this study. High affinity of IgG antibody to human peptides antigen was shown in a previous study [42,43,44]. IgG type antibody contained two moles of binding sites for each mole of antibody, thus, a maximum of 1 to 2 mole ratio of antibody to antigen could be reached by using IgG antibody for human peptides. Therefore, the highest concentration of β-amyloid 42 antigens was determined based on the mole ratio of antibody to antigen. Detectable concentration of β-amyloid 42 in this study ranged from 0.0625 µg/mL to 0.5 µg/mL.

### 4.2. Detection of β-Amyloid 42 in PBS Solution

**Figure 1:**
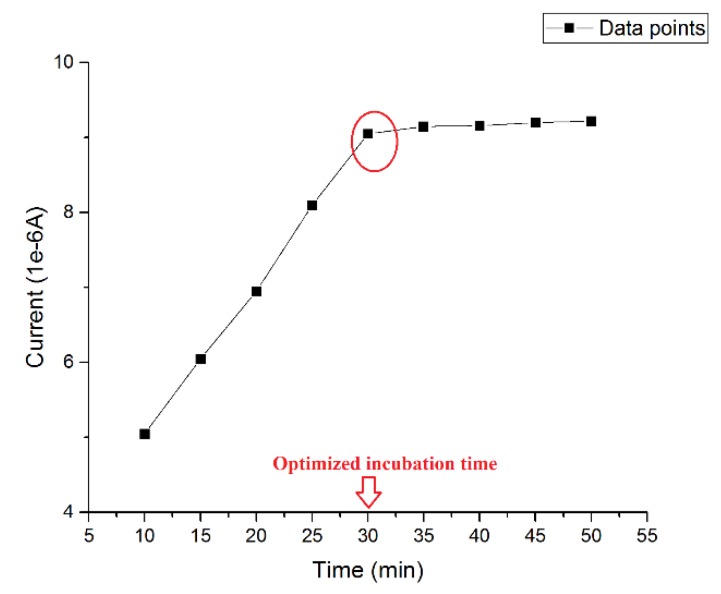
Optimized incubation time of β-amyloid 42.

The incubation time of β-amyloid 42 was pivotal to the success of the detection. An ideal incubation time should produce the strongest current signal without interference from aggregation of antigen during the incubating process. Thus, the optimized incubation time was investigated using a 0.5 µg/mL β-amyloid 42 antigen solution and then the DPV measurement was conducted at the presence of 5 mM of K_4_Fe(CN)_6_ and K_3_Fe(CN)_6_ on the electrode surface at different incubation times. The volume of both the β-amyloid 42 solution and K_4_Fe(CN)_6_ and K_3_Fe(CN)_6_ redox solution on the sensor were 20 µL. Figure 1 shows the different current outputs based on different incubation times. A relatively high signal was produced after 30 min incubation at room temperature. For an incubation time longer than 30 min, the width of the DPV curve became larger, which may indicate an inhomogeneous distribution of surface particles due to aggregation of β-amyloid 42. The results of this study were consistent with previous research reported [27,45,46]. Therefore, an incubation time of β-amyloid 42 antigen for 30 min was chosen for this study.

**Figure 2:**
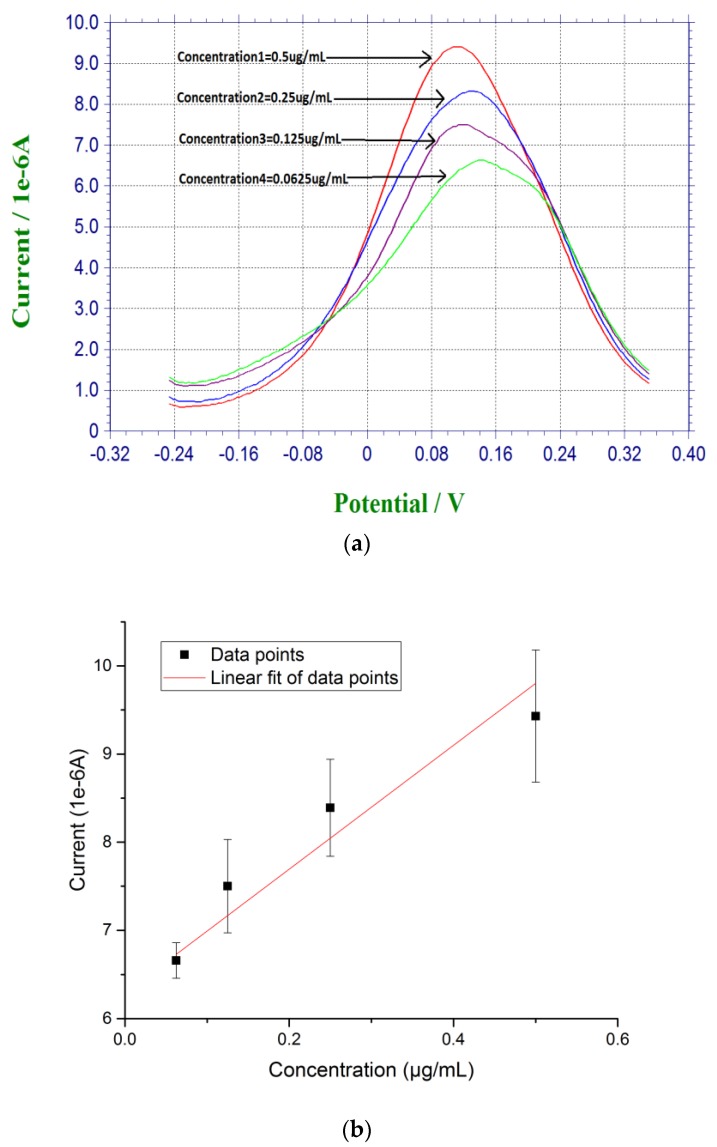
(**a**) DPV measurement of β-amyloid 42 antigens with concentrations ranging from 0.0675 µg/mL to 0.5 µg/mL in PBS; and (**b**) Calibration curve based on the DPV measurement of β-amyloid 42 in PBS (*n* = 3).

For DPV measurement, 20 µL of prepared β-amyloid 42 antigen solution was placed on top of each biosensor for 30 min at room temperature. After incubation for 30 min, the biosensor was rinsed by PBS. 20 µL of a redox probe couple solution, containing 5 mM each of K_4_Fe(CN)_6_ and K_3_Fe(CN)_6_, was then added on top of the biosensor after the incubation of the antigen of β-amyloid 42, and DPV measurement was then made. Based on the radicalization of β-amyloid 42 thin film after incubation, larger concentration of β-amyloid 42 sample produced a higher conductivity or lower resistance on the electrode surface with the use of redox probe couple [27,45]. Figure 2a shows the DPV measurements of β-amyloid 42 ranging from 0.0675 µg/mL to 0.5 µg/mL with a decreasing sequence of current output. Figure 2b shows the calibration curve of the DPV measurements based on the concentration of β-amyloid 42 antigen and the corresponding current outputs with a linear fit equation of Y = 7.74X + 6.28. The R square value of 0.85 shows a high reproducibility of the biosensor with *n* = 3.

### 4.3. Detection of β-Amyloid 42 in Human Serum

**Figure 3:**
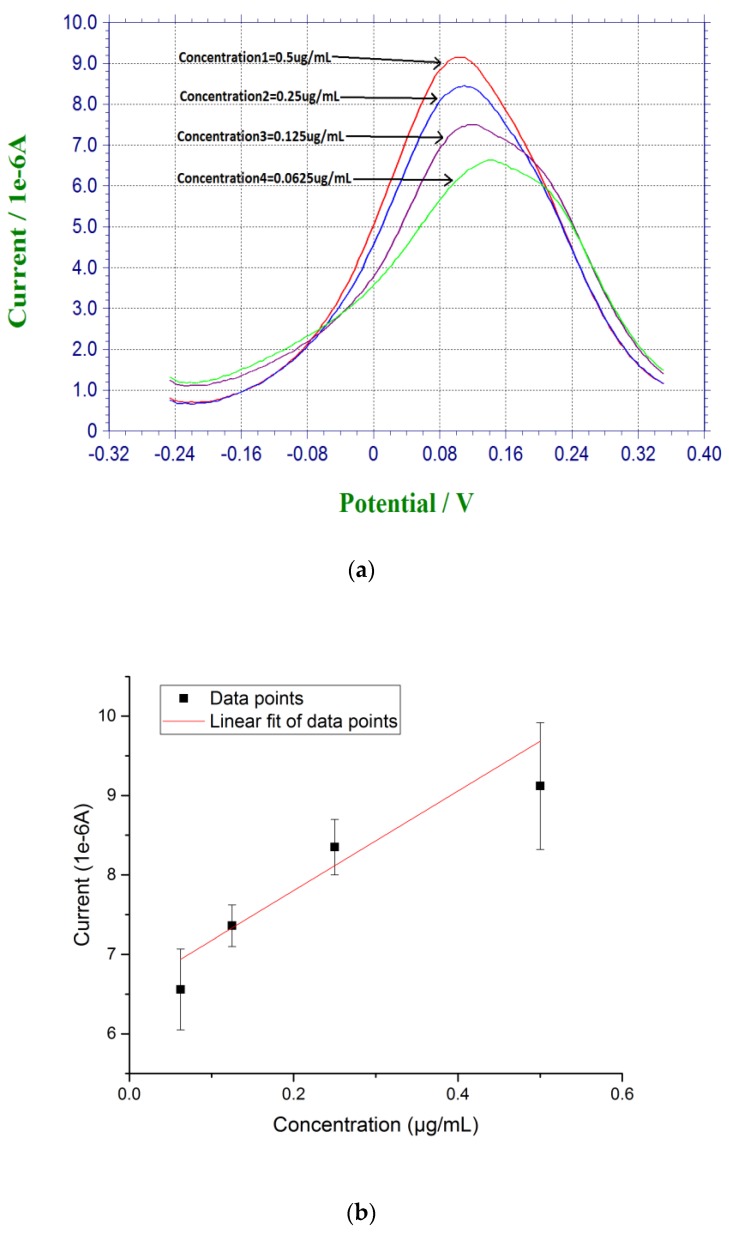
(**a**) DPV measurement of β-amyloid 42 antigens with concentrations ranging from 0.0675 µg/mL to 0.5 µg/mL in human serum; and (**b**) Calibration curve based on the DPV measurement of β-amyloid 42 in human serum (*n* = 3).

Measurements based on β-amyloid 42 in human serum sample were also conducted. Instead of diluting the pre-dissolved DMSO β-amyloid 42 sample in PBS, human serum was used for further dilution for testing. The β-amyloid 42 antigen concentrations range was 0.0675 µg/mL to 0.5 µg/mL. First, 20 µL of the prepared β-amyloid 42 solution in human serum was placed on top of the biosensor for 30 min at room temperature. The sensor was rinsed with PBS before testing. Similar to the DPV measurement in PBS solution testing described above, 20 µL of a redox probe couple solution, containing 5 mM each of K_4_Fe(CN)_6_ and K_3_Fe(CN)_6_, was then added on top of the biosensor after the incubation of the antigen of β-amyloid 42 and DPV measurement was then made. A calibration curve of Y = 6.55X + 6.70 with R square value of 0.94 showed a good reproducibility of the biosensors in human serum testing media with *n* = 3 as shown in Figure 3.

### 4.4. Interference Study of β-Amyloid 42 Sensor Against Tau Protein Antigen

**Figure 4:**
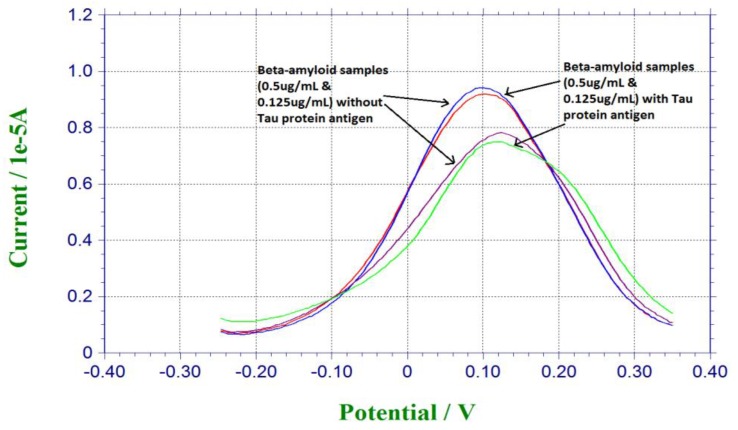
Interference study based on Tau protein antigen.

Tau protein antigen was also a significant biomarker for neuro-degenerative disease [1,2,3]. Tau protein was investigated in another study conducted with a similar platform binding technique [32]. In order to evaluate the selectivity of our biosensor, Tau protein antigen was used as the potential interference chemical. For preparation of the antigen solution, 0.125 µg/mL and 0.5 µg/mL of β-amyloid 42 antigen solutions were mixed with the same amount of Tau protein antigen. 20 µL of the mixture of β-amyloid 42 antigen and Tau protein antigen was dropped on the sensor for incubation for 30 min, and then 20 µL of 5 mM redox solution of K_4_Fe(CN)_6_ and K_3_Fe(CN)_6_ was dropped on the sensor for testing. Figure 4 shows the responses from pure beta-amyloid sample and mixed Tau protein and amyloid sample. Similar current outputs were observed from the DPV measurements, indicating the Tau protein amyloid sample. Similar current outputs were observed from the DPV measurements, indicating that Tau protein antigen did not contribute to the DPV measurement of β-amyloid 42 antigen This interference study demonstrated the high selectivity of our β-amyloid 42 biosensor.

## 5. Conclusions

A cost-effective, single-use, in vitro biosensor for the detection of a biomarker of neuro-degenerative disorder, β-amyloid 42, has been designed, manufactured, and evaluated in phosphate-buffer saline and in undiluted human serum. The bio-recognition mechanism of this biosensor was based on the effect of the interaction between antibody and antigen of β-amyloid 42 to the redox couple probe of K_4_Fe(CN)_6_ and K_3_Fe(CN)_6_. Differential pulse voltammetry (DPV) was the transduction mechanism and used as the measurement technique. Measurements of β-amyloid 42 antigens in both 0.1 M PBS and undiluted human serum over the concentration range of 0.0675 µg/mL to 0.5 µg/mL showed excellent results and good linearity of the calibration curves. The antibody concentration of β-amyloid 42 used was 18.75 µg/mL. Tau protein antigen was used in the interference study, and this β-amyloid 42 biosensor demonstrated excellent specificity, as shown without interference by Tau protein antigen. This biosensor platform technology can be further optimized and applied to detect other biomarkers of neuro-degenerative disorders.
